# Evaluation of Structural Behavior in the Process Dynamics of Oleogel-Based Tender Dough Products

**DOI:** 10.3390/gels8050317

**Published:** 2022-05-19

**Authors:** Anda E. Tanislav, Andreea Pușcaș, Adriana Păucean, Andruța E. Mureșan, Cristina A. Semeniuc, Vlad Mureșan, Elena Mudura

**Affiliations:** Food Engineering Department, Faculty of Food Science and Technology, University of Agricultural Sciences and Veterinary Medicine Cluj-Napoca, 3-5 Calea Mănăștur Street, 400372 Cluj-Napoca, Romania; anda.tanislav@usamvcluj.ro (A.E.T.); andreea.puscas@usamvcluj.ro (A.P.); andruta.muresan@usamvcluj.ro (A.E.M.); cristina.semeniuc@usamvcluj.ro (C.A.S.); elena.mudura@usamvcluj.ro (E.M.)

**Keywords:** oleogelators, pastry products, rheology, hardness, margarine replacer

## Abstract

The current trend is represented by replacing solid fats with structured liquid oil while maintaining the plastic properties of food products. In this study, the behavior of refined sunflower oil structured with various agents (carnauba wax-CRW, *β*-sitosterol:beeswax-BS:BW, *β*-sitosterol:lecithin-BS:LEC, and glycerol monostearate-GM) was evaluated in the process dynamics of oleogel-based tender dough products. The oleogel with the mixture of *β*-sitosterol:beeswax (OG_BS:BW) displayed the highest capacity to retain oil inside the matrix with a percentage of oil loss as low as 0.05% and also had a significantly higher hardness (6.37 N) than the reference, a commercial margarine (MR—3.58 N). During cooling from 90 to 4 °C, the increase in oleogel’ viscosity results from oleogelator’s liquid–solid phase transition. As demonstrated by the frequency sweeps performed, storage modulus G′ was higher than loss modulus G″, no cross-over points were observed, and the strongest gel network was for the oleogel with glycerol monostearate (OG_GM). Regarding the dough, the sample prepared using the oleogel with carnauba wax (D_CRW) showed the strongest hardness (92.49 N) compared to the reference (D_MR—21.80 N). All the oleogel-containing doughs had elastic solid-like behavior. The samples with margarine (D_MR) and the mixture of *β*-sitosterol:lecithin (D_BS:LEC) presented the lowest value of both moduli of G’ and G” during the frequency sweep. The biscuits formulated with commercial margarine (B_MR) registered a hardness of 28.74 N. Samples with oleogels showed a specific tenderness for tender dough products, thus being suitable for this type of product (11.22–20.97 N).

## 1. Introduction

Oils and fats are important sources of energy, solvent mediums for vitamins and bioactive compounds, and imprint functionality, texture, and palatability in food products [1].

Solid fats are composed of fat crystal networks consisting mainly of *trans* and saturated fats, and their elastic properties are relevant in obtaining texture, spreadability, and unctuosity [2]. To avoid major differences in texture, oil separation, and other problems caused by saturated fat substitution, new methods for oil structuring should be used to develop unsaturated fat alternatives with properties and functions comparable with those of saturated fats [3].

The Food and Drug Administration removed *trans* fats from the list of substances generally recognized as safe (GRAS) in 2015 and completely banned the use of partially hydrogenated oils in foods since January 2020. The World Health Organization recommends a consumption of <1% *trans* fats (2.2 g/day at an intake of 2000 kcal), <10% of the total daily energy to result from saturated fats. The European Commission, through the Regulation (EU) 2019/649, has set the maximum level for trans fats added in food as follows: “The content of *trans* fat, other than *trans* fat naturally occurring in fat of animal origin, in food intended for the final consumer and food intended for supply to retail, shall not exceed 2 g per 100 g of fat” [4]. Due to those provisions, the food industry has replaced partially hydrogenated fats with saturated fats, the most widely used being palm oil [5,6] and fully hydrogenated vegetable oils (do not contain *trans* fats) [2,7].

Shortening contains a mixture of solid fat crystals and liquid oil. The solid fat content offers a three-dimensional crystalline matrix that has the role of binding the liquid phase and providing plasticity to the dough. The consistency, crystalline structure, and melting characteristics affect the shortening function depending on the purpose of the application for which it is designed [8]. In the case of biscuits, large amounts of sugar and fat are used, while the moisture content remains reduced, so the shortening used must be in a solid or semi-solid state at room temperature to ease the handling of the dough in the manufacturing process [9]. The type of shortening used must have plastic properties that are characterized by the solid fat content (a high value does not lead to a volume of oil required for sufficient aeration, while a low value is not able to retain enough air bubbles during homogenization) [10].

Oleogelation is a useful alternative to structure vegetable oils without the use of *trans* or saturated fats and oleogels are defined as gels of organic solvents. This technique modifies the physical structure of vegetable oils based on the physicochemical properties of the oleogelators. The possibility of gelling a large amount of solvent (oil type) using a small amount of gelling molecules (approximately 5–10%) makes oleogelation an efficient way for structuring oil in the absence of saturated fats or with a reduced content of this [11]. According to the FDA, carnauba wax and glycerol monostearate can be used in foods at levels not exceeding a good manufacturing process [12,13]. The EFSA concluded that the use of beeswax as an additive is not of safety concern [14].

Oleogelators transform liquid oils into thermoreversible and three-dimensional gel networks with solid properties. The structural and physical properties of the formed oleogels can be influenced, depending on the application by various factors, such as type and concentration of oleogelators, type of vegetable oils (determines the critical gelling concentration of a gel, attributed to its solubility), and processing conditions [15].

Oleogels have been studied and used to replace shortening in bakery products in order to reduce the level of *trans* and saturated fats. The saturated fats from the biscuits were reduced by mixing an oleogel based on candelilla wax with commercial shortening in the ratio of 30:70 and 60:40 and the effect on the hardness, extensibility, and texture of biscuits was evaluated. Dough samples with oleogel were softer than those with oleogel-shortening mixtures, which were slightly softer than dough containing just shortening. Doughs containing canola oil were much softer than those containing wax-based oleogels, and the hardness of the dough increased with the concentration of wax. The extensibility of the dough was lowest for samples with canola oil, and that for the dough containing shortening was closest to the samples containing oleogel-shortening mixtures. Biscuits prepared with 40% oleogel and 60% shortening had a similar texture to biscuits prepared only with shortening [16]. In another study, canola oil structured with candelilla wax (3% and 6% *w*/*w*) was formulated and added to cookies where the unsaturated fatty acid content increased to 92% as opposed to the control samples made with shortening (47.2%) [17], and when rice bran wax/beeswax (10% *w*/*w*) and sunflower oil oleogels were used in cakes, the content of unsaturated fatty acids increased to 82–86% and the level of saturated fatty acids decreased to 14–17% compared to the control sample, which contained only shortening (58%) [18].

The textural, sensory, and stability properties of biscuits containing oleogels formulated with 5% sunflower wax and beeswax were studied. Commercial shortening was used as the control sample. Differences in color were observed between the surface of the biscuits containing different waxes. The shortening samples developed a less aerated structure and were harder than oleogel samples (due to the higher presence of liquid oil). In terms of sensory attributes, such as appearance and aroma, it has been shown that beeswax and sunflower wax can produce biscuits with equal or higher performance compared to those made exclusively with commercial shortening. Hedonic consumer data have indicated that oleogel-based biscuits are preferred and better accepted [19]. In the formulation of oleogels, not only small molecules such as waxes can be used, but modified cellulose is also a commonly used organogelator. Sunflower oil was structured with hydroxypropyl methylcellulose and used in four different proportions (25%, 50%, 75%, and 100% *w*/*w*) to replace the shortening from muffins. The consistency index decreased when the oleogels had a lower content of hydroxypropyl methylcellulose. The loss of specific volume was significant only when the shortening was replaced with oleogel in 75% and 100%. The structure of the air cell was less compact and denser when up to 50% oleogel was used [20]. Preventing volume loss is one of the technical challenges when replacing solid fats with oleogels. An oleogel with canola oil and carnauba wax was prepared to replace solid fat in aerated baked goods. The porosity index and fragmentation decreased as the oleogel content increased, and the specific volume of the cakes was maintained by replacing the shortening with oleogel up to 50% [21].

This research aims to replace the conventional saturated fats by developing suitable oleogels based on unsaturated lipids and assessing their performance in the manufacturing process of tender dough biscuits. As research about the behavior of oleogel during the food obtaining process is limited, we decided to evaluate the behavior of oleogels during each technological step (oleogels-doughs-biscuits) of the tender dough products process. As good results were obtained with a 100% replacement of baker’s margarine for wax-based oleogels, the study was extended to other structurants. Biscuits are widely consumed snacks, and there are already improved options (digestive, with various flours), but replacing saturated fats is a step to make this product suitable for a larger number of consumers.

## 2. Results and Discussion

### 2.1. Oleogel Characterization

**Figure 1:**
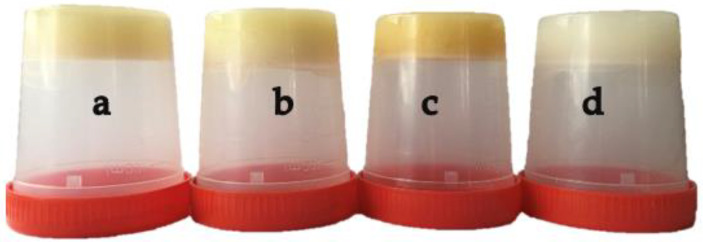
Visual appearance of (**a**) oleogel with carnauba wax (OG_CRW), (**b**) oleogel with *β*-sitosterol:beeswax (OG_BS:BW), (**c**) oleogel with *β*-sitosterol:lecithin (OG_BS:LEC), and (**d**) oleogel with glycerol monostearate (OG_GM).

The visual appearance of oleogels is depicted in Figure 1. All the oleogels prototypes formulated with refined sunflower oil and various structuring compounds led to the formation of a stable gel. No syneresis or leakage was observed while inverting the recipients at room temperature for one hour. Due to the type of oleogelators used, the direct structuring procedure was adequate to obtain these structured lipid mixtures. During the cooling process, the oleogelators form a three-dimensional and thermoreversible gel network that captures the liquid oil, thus changing the mixture into a semi-solid liquid called oleogel [22].

The results from textural profile analysis, color, and oil binding capacity for the oleogels are presented in Table 1.

#### 2.1.1. Textural Profile Analysis (TPA)

Hardness (N) is the maximum value of the first compression cycle and, from a sensory point of view, the maximum force required to compress the product between the molars. Oleogel with the mixture of *β*-sitosterol:beeswax (OG_BS:BW) had a significantly higher hardness (6.37 N), a property attributed to the superior structuring capacity offered by these oleogelators. For the other samples, including the control sample (MR), the hardness value was similar, indicating that the liquid oil matrix was adequately structured and, from this point of view, oleogels may be an alternative to saturated fats. Harder oleogels can adversely affect the way dough is processed for bakery products [23]. The oleogel hardness is influenced by storage temperature, oleogelator composition, and the degree of crystallinity [24]. Kupiec et al. [25] obtained oleogels from rapeseed oil and 5% beeswax and reported similar results in term of firmness (5.60 N).

Adhesiveness (mJ) is the force required to overcome the attraction forces from the surface of the product when it comes into contact with a surface (tongue, teeth). Adhesiveness is the negative area obtained during the first cycle and influences the processing and rolling characteristics of the doughs. Among all samples, commercial margarine (MR) had the highest value for this parameter (2.10 mJ), a characteristic transposed in the technological process where the dough obtained with it was the most difficult to roll. For oleogel samples, the adhesiveness was lower, in the range of 0.55–1.55 mJ. The proportion of gelling agents influenced the adhesiveness as follows: OG_BS:LEC with the highest proportion of structurants (16%) showed a higher adhesiveness in contrast to the rest of the samples where 10% structuring agents were used. In the study conducted by Pandolsook and Kupongsak [26], they reported an increase in adhesiveness with the proportion of structuring agents (3% rice bran wax compared to 9% rice bran wax).

Cohesiveness indicates the strength of the product internal bonds (the higher the value, the greater the cohesion). Textural data provide the ratio between the positive force of the second compression cycle and the force required in the first compression cycle, a critical quality parameter for dough tender alternatives because it affects the texture of the finished product. The cohesiveness value of the oleogels was lower (0.05–0.21) as opposed to the MR (0.59). According to Ye et al. [27], the lower the cohesiveness, the finer the texture, a statement correlated in the present study by the textural analysis of biscuits. As with the adhesiveness results, the higher amount of oleogelators used for OG_BS:LEC led to a higher cohesiveness. In a study on canola oil and carnauba wax (10%) oleogels, the results obtained for cohesiveness are similar to those of our study (0.20). In the same study, oleogel with 10% beeswax reported a cohesiveness of 0.33, which indicates the effect that *β*-sitosterol in combination with wax has on decreasing cohesiveness (0.05 for OG_BS:BW in our study) [28].

#### 2.1.2. Oil Binding Capacity

The ability of oleogels to retain oil inside the matrix provides information about the stability of the gel, the effectiveness of the crystal network structuring [29], and the potential for application in food products [30].

According to the literature, the strongest oleogel must have the best oil binding capacity [29,31], and also the ratio of the gelling agents is an important factor [32]. Thus, the OG_BS:BW had a percentage of loss oil of 0.05%. This result correlated with the textural profile analysis, where this oleogel presented the highest hardness value (6.37 N). Oleogel with *β*-sitosterol:lecithin (OG_BS:LEC) caused higher values of oil expelled during centrifugation (2.77%), although the percentage of structurants in this system was 16%. Following the oleogel structuring with different types of waxes and lecithin, Okuro et al. [29] concluded that lecithin (1.75–7.00%) improves the mechanical properties of gels and increases the oil binding capacity [29]. In the present study, the prototype formulated with lecithin represented 3.2% of the total amount of structurants.

Although in terms of hardness, oleogel with glycerol monostearate (OG_GM) and oleogel with carnauba wax (OG_CRW) showed similar values (2.46 N and 2.54 N), the oil loss was lower for the first sample (2.53%) as opposed to the one structured with carnauba wax (3.15%). Thakur et al. [33] reported reduced oil losses for oleogel with 10% carnauba wax.

The results indicate that the crystalline phase was sufficiently structured to develop a network that maintained the oil in a gel-like structure. All formulated prototypes optimally structured the refined sunflower oil, with the oil losses compared to the initial mass of oleogel being reduced (<3.2%). The ability of a compound to gel a solvent is considered to be a balance between the solubility and insolubility of the gelling agent in the solvent (it must not be insoluble but not very soluble). Thus, it should be relatively insoluble to crystallize into central elements, which gives structure but is also partially soluble to interact with solvent molecules [34].

#### 2.1.3. Oleogel Rheology

**Figure 2:**
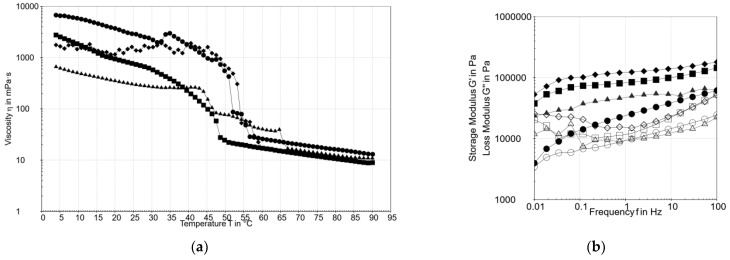
(**a**) Viscosity obtained during cooling from 90 to 4 °C at 1 °C/min. 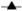 OG_CRW, 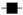 OG_BS:BW, 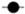 OG_BS:LEC, 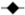 OG_GM. (**b**) Frequency sweep at a strain of 0.01% and 20 °C. 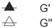 OG_CRW, 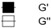 OG_BS:BW, 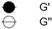 OG_BS:LEC, 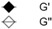 OG_GM.

The viscosity changes associated with oleogelation with different structuring compounds during cooling from 90 to 4 °C were studied (Figure 2a). The cloud point for each oleogel was: 50.6 °C for OG_BS:BW, 57.1 °C for OG_BS:LEC, 60.4 °C for OG_GM, and 67.4 °C for OG_CRW. After this point was reached, a sudden increase in viscosity appeared because the nucleation process started. Wijarnprecha et al. [35] reported similar results for oleogels formulated with rice bran oil and rice bran wax in different concentrations. The lowest temperature at which the oleogel can be poured under the prescribed conditions is represented by the pour point: 35.5 °C for OG_BS:BW, 35.3 °C for OG_BS:LEC, 45.3 °C for OG_GM, and 42.6 °C for OG_CRW.

Frequency sweeps results showed that G′ (storage modulus) was higher than G″ (loss modulus), and both slowly increased with frequency (Figure 2b). No cross-over points were observed for these oleogels and the loss factor tan δ was <1. OG_BS:LEC presented the weakest gel network but a greater ability to resist deformation at the 0.01% strain used. The structure of the gels network from the strongest to the weakest was: OG_GM > OG_BS:BW > OG_CRW > OG_BS:LEC. Similar results were obtained for oleogels with monoglycerides [31], carnauba wax [21,23], and beeswax [36].

#### 2.1.4. Color Determination of Oleogel Samples

The color of the oleogels was different on visual inspection. The color properties of oleogels influence the possibility of application in the food industry. The range of color parameters for structured lipids, stored at refrigeration temperature, was: 73.16–84.18 for L*, 11.66–32.85 for a*, and 19.15–33.91 for b*.

All samples were opaque and yellow (in various shades). The OG_BS:LEC was noticeable because of its slightly darker color; as a result, it had the lowest brightness index (L*—73.16), and it was identified by the strongest color in the red spectrum (a*—32.85) and the most intense yellow color (b*—33.91). On the other hand, MR was characterized by a light color, which was identified by the lowest parameters for red and yellow (a*—11.66 and b*—19.15, respectively) and the highest brightness (L*—84.18). Similar values were obtained for the OG_GM, which showed the smallest color difference compared to the control sample (ΔE—12.74). Paciulli et al. [37] reported that the color difference, ΔE, is not noticeable when it is less than 3. In this study, high ΔE values (12.74–28.08) were reported for oleogels, indicating that color differences between samples were detectable.

**Table 1:** Textural properties, color, and oil structuring capacity of oleogel samples containing different oleogelators.

Parameters	OG_GM	OG_CRW	OG_BS:BW	OG_BS:LEC	MR
**Texture profile analysis**					
Hardness (N)	2.46 ^B^ ± 0.25	2.54 ^B^ ± 0.63	6.37 ^A^ ± 0.18	2.93 ^B^ ± 0.25	3.58 ^B^ ± 0.32
Adhesiveness (mJ)	0.95 ^AB^ ± 0.49	0.55 ^B^ ± 0.21	1.20 ^AB^ ± 0.28	1.55 ^AB^ ± 0.49	2.10 ^A^ ± 0.00
Cohesiveness	0.08 ^B^ ± 0.01	0.12 ^B^ ± 0.01	0.05 ^B^ ± 0.03	0.21 ^B^ ± 0.12	0.59 ^A^ ± 0.00
**Color**					
L*	77.16 ^AB^ ± 5.47	75.70 ^AB^ ± 4.92	79.28 ^AB^ ± 3.50	73.16 ^B^ ± 2.47	84.18 ^A^ ± 2.66
a*	22.09 ^B^ ± 3.20	24.74 ^B^ ± 3.66	26.32 ^AB^ ± 5.65	32.85 ^A^ ± 2.97	11.66 ^C^ ± 2.09
b*	21.23 ^C^ ± 2.30	31.13 ^AB^ ± 1.87	30.37 ^B^ ± 1.65	33.91 ^A^ ± 0.64	19.15 ^C^ ± 0.81
ΔE	12.74	19.66	19.10	28.08	-
**Oil binding capacity**					
Oil loss (%)	2.53 ^A^ ± 0.36	3.15 ^A^ ± 0.37	0.05 ^A^ ± 0.00	2.77 ^A^ ± 1.55	-

Values are expressed as mean ± standard deviation. For each characteristic, identically superscript capital letters indicate no significant differences (*p* > 0.05) between samples. ΔE indicates color differences of the samples in comparison to MR.

### 2.2. Dough Texture, Rheometric Analysis, and Color Determination

The final recipe was optimized to be suitable for 100% replacement of the commercial margarine. During dough preparation, the sugar is initially mixed with the margarine and the egg to capture the structured air in the liquid phase of the fat. Then, flour is added with a minimal mixture to achieve a minimum development of gluten [9].

The results from textural analysis and color determination for dough samples are presented in Table 2.

Hardness (N) is defined as the force required to achieve a certain deformation. The hardness value for the dough formulated with OG_CRW (D_CRW—92.49 N) was significantly higher, compared to the dough obtained with MR (D_MR—21.80 N). Because the resting process of the samples was performed at the refrigeration temperature, a higher hardness value was expected for the control dough. The hardness of the doughs obtained with the rest of the structured lipid prototypes showed similar values and was also higher than for the control sample.

The springiness index indicates the recovery properties of the dough (a value of 1 indicates a completely elastic material and 0 indicates a completely viscous material). In practice, this index shows us the behavior that the dough will have during rolling and shaping and is correlated with adhesiveness. Thus, the control sample (D_MR) showed the highest value of adhesiveness and the most elastic behavior (4.90 mJ and 0.23), compared to the sample made with OG_BS:LEC (D_BS:LEC—2.05 mJ and 0.11). In the practical manufacturing process of biscuits, the prototypes made with the *β*-sitosterol mixture (D_BS:LEC, D_BS:BW) performed with the best behavior during rolling and modeling operations. In the meantime, the D_MR was difficult to process due to its high adhesiveness, and the doughs with glycerol monostearate (D_GM) and carnauba wax (D_CRW) were found to not be compact enough.

Resilience is the measurement of how a product is able to recover after deformation in relation to the applied speed and forces. In terms of cohesion and strength (“instantaneous elasticity”), they showed opposite trends, indicating that a dough more cohesive has a lower recovery capacity after being subjected to stress. While the cohesiveness showed the same trend for the samples made with oleogels, it increased for the sample made with margarine; the cohesion value for this dough was almost triple.

**Figure 3:**
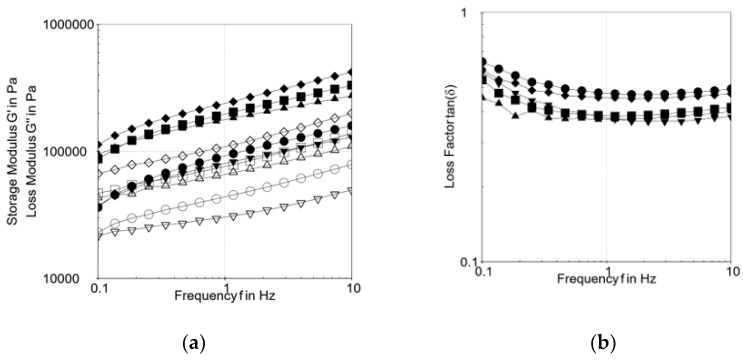
(**a**) Changes in the dynamic viscoelastic properties of oleogel and margarine doughs: G’ and G”. 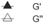 D_CRW, 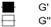 D_BS:BW, 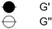 D_BS:LEC, 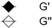 D_GM, 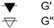 D_MR. (**b**) Changes in the dynamic viscoelastic properties of oleogel and margarine doughs: tan δ. 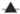 D_CRW, 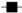 D_BS:BW, 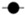 D_BS:LEC, 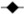 D_GM, 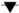 D_MR.

The effects of margarine replacement with oleogels on the viscoelastic properties of tender dough were measured as a function of frequency. All the dough samples exhibited higher values of G’ (storage modulus) than G” (loss modulus) in the frequency range tested (Figure 3a). The loss factor tan δ initially decreased with the frequency, and then stabilized and slowly increased in the end (Figure 3b), and both moduli (G′ and G″) increased with frequency, showing a frequency dependence. All the dough samples had elastic solid-like behavior because the values of tan δ were less than 1, indicating this fact. Jung et al. [38] formulated blends between rice bran oil-candelilla wax oleogel and butter (100:0, 75:25, 50:50, 25:75, 0:100) and introduced them in sweet pan bread. They obtained similar results for the dynamic viscoelastic measurements of the dough samples. The D_MR and D_BS:LEC showed the lowest values of G’ and G” compared with those formulated with the rest of oleogels. The results are correlated in terms of strongest dough to those obtained in this study at the oleogel rheology analysis.

Regarding the color determination, although the OG_BS:LEC showed the lowest brightness index, in the dough samples, the L* value was highest (70.28), followed by the D_MR (68.01), which previously presented the highest brightness index. The values in the red spectrum a* oscillated between 5.70 and 6.98, while, in the yellow spectrum b*, it oscillated between 20.89 and 24.22. The D_CRW, D_BS:BW, and D_BS:LEC had similar tones in the red spectrum. The highest color difference was for the dough obtained with OG_CRW (10.57), while the rest of the samples showed insignificant differences: D_GM—3.16, D_BS:BW—4.23, and D_BS:LEC—2.83 (where a value below 3 indicates that the difference is not visible with the human eyes [37]).

### 2.3. Biscuits Texture Analysis and Color Determination

Textural properties and color parameters of biscuits are represented in Table 3.

Hardness is considered an important characteristic of the quality of biscuits because it affects the acceptability of consumers and the desire to buyback. The hardness values were between 11.22 N for biscuits with OG_BS:BW (B_BS:BW) and 28.74 N for the commercial margarine sample (B_MR). Although the prototype with glycerol monostearate (B_GM) registered a hardness (20.97 N) similar to the B_MR, by simple organoleptic analysis, the sample showed a specific tenderness, similar to the rest of samples made with oleogels. On the other hand, Li et al. [39] reported a significantly higher hardness for cookies obtained with 6% beeswax (47.50 N) and monoacylglycerol (40.90 N), and also Yilmaz and Ogutcu [19] for cookies with beeswax (31.85 N) and commercial bakery shortening (47.13 N). The differences between samples can be attributed to the different recipes and technological parameters used.

Fracturability [N] refers to the easiness with which the product will break, directly correlating with the hardness value. All samples showed similar fracturability and hardness, except the B_CRW. Jan et al. [40] showed that the decrease in the hardness of the samples may be due to the tenderized effect of fat and the encapsulation of flour particles by fat, thus isolating the particles from each other and making them more detachable.

The color surface of the biscuits represents an important indicator of the degree of baking and influences the consumers’ perception regarding the aspect of the finished product [40]. The instrumental brightness (L* values) of the biscuits revealed that the lighter surface color was for the sample formulated with MR (73.62), and the darker color was for the sample obtained with OG_GM (68.21). According to Kaur et al. [41], these values can be explained by the Maillard browning reactions occurring during baking. The color differences between oleogel and margarine samples were visible (values are bigger than 3 [37]) and were in the range of 5.49–7.41. Similar results were obtained by Onacik-Gur and Zbikowskain [42] in the study on biscuits formulated with oleogel with monoacylglycerol (L*—71.44, a*—2.52, b*—24.74) and beeswax (L*—72.87, a*—1.68, b*—24.41) and by Li et al. [43] on cookies with oleogel with monoacylglycerol (L*—70.00, a*—9.3, b*—24.30).

## 3. Conclusions

In this study, the evaluation of the structural behavior during the technological process was performed for biscuits obtained with four different oleogels and compared with baking margarine. Due to the health issue associated with saturated fats, consumers are more focused and choose food products formulated with alternatives to conventional fats. The hardness of the oleogels was correlated with their ability to structure the oil in the gel matrix, so that the strongest gel, with a 10% mixture of *β*-sitosterol:beeswax, showed the lowest oil loss compared to other fat systems where higher oil losses occurred when the hardness was lower. However, the oil losses for all samples were below 3.2%, which showed that the concentration of gelling agents (10 and 16%) was adequate to form a stable gel and to structure the network efficiently. The color of the oleogels was correlated with the doughs and biscuits samples obtained, and it is a very important quality attribute that considerably influences consumers’ perception of the desire to buy this type of product: regarding the doughs, the smallest color difference (2.84) was for the *β*-sitosterol:lecithin sample, which also showed a hardness value similar to the baker’s margarine sample. The rheological analysis of the doughs showed that the storage modulus G’ and the loss modulus G’’ were similar to those of the oleogels and the structure from the strongest to the weakest was: D GM > D BS: BW > D CRW > D BS: LEC. The biscuits formulated with oleogels were suitable for the category of tender dough products due to the finer texture offered by the addition of oleogels, especially for the samples obtained with *β*-sitosterol mixtures and carnauba wax.

In addition to meeting the need for unsaturated fatty acids, oleogels have the potential to create food products with desired physical properties. Therefore, further studies such as sensory evaluation and oxidative stability can be conducted in order to observe consumers’ preferences and the shelf life of this product.

## 4. Materials and Methods

### 4.1. Materials

The organogelators used were carnauba wax (2442L) and beeswax (8109) from Kahlwax GmbH & Co. KG., *β*-sitosterol (approx. 10% campesterol, approx. 75% beta-sitosterol) from Acros Organics, purified glycerol monostearate from ThermoFisher GmbH, and soy lecithin (non-genetically modified) from ThewArnott. The waxes are natural according to the description provided by the producers and are stored under refrigerated conditions (0–4 °C). Refined sunflower oil and the raw materials used to make the biscuits (white wheat flour, powdered sugar, and eggs) were purchased from a local store. Commercial margarine consisting of palm, sunflower, rapeseed, and coconut vegetable oils in varying proportions (60% fat), water, mono- and diglycerides of fatty acids, salt, sorbic acid, flavor, citric acid, dye, and vitamins were produced in Romania and purchased from a local distributor.

### 4.2. Oleogel Preparation

The oleogels were prepared by the direct method using refined sunflower oil and various oleogelators. Gelling agents and their concentration were selected after prior consultation with the literature and above the critical gelation point [33,44,45,46]. The total concentration of the structurants was 10% (*w*/*w*) for carnauba wax, glycerol monostearate, and the mixture of *β*-sitosterol:beeswax (2:8), and 16% (*w*/*w*) for the mixture of *β*-sitosterol:lecithin (8:2). The mixtures were heated at 90 °C using a magnetic stirrer (200 rpm) to ensure melting and complete dissolution. The clear oil dispersions were crystallized immediately after melting at ~4 °C for 24 h. These stock oleogels were analyzed and used for the production of doughs and biscuits. The samples were coded as: oleogel with carnauba wax (OG_CRW), oleogel with glycerol monostearate (OG_GM), oleogel with *β*-sitosterol:beeswax (OG_BS:BW), and oleogel with *β*-sitosterol:lecithin (OG_BS:LEC).

### 4.3. Oleogel Characterization

#### 4.3.1. Texture Profile Analysis (TPA)

The Brookfield CT3 texture analyzer (Brookfield Engineering Labs, Middleboro, MA, USA) and the method described by Hwang et al. [47] were used to evaluate the textural profile of the samples (30 mm height × 45 mm diameter). The method consists in penetrating the samples (in two cycles) with the TA18 spherical compression probe that is attached to a 10 kg compression cell. The compression of the samples was performed at a depth of 5 mm from the sample surface, with a speed of 1 mm/s, with the compression probe being extracted with the same speed. The instrument records the maximum force during compression, expressed as firmness (N) as a function of time (s). The main parameters followed in the textural analysis were: hardness, adhesiveness, and cohesiveness. The samples were stored at 4 °C and analyzed directly from this temperature.

#### 4.3.2. Oil Binding Capacity (OBC)

The oil binding capacity was achieved by subjecting the oleogel samples to the centrifugal force according to the method described by Okuro et al. [29]. Approximately 10 g of each oleogel type was introduced after melting into conical tubes with a screw cap, Falcon type (50 mL), crystallized at 4 °C and placed in the Hettich Universal 320R centrifuge (Andreas Hettich GmbH & Co. KG, Tuttlingen, Germany). These were centrifuged for 30 min at a maximum speed of 9000 rpm corresponding to a relative centrifugal force of 13.1 × 10^3^ g. To avoid oleogel melting, the temperature during centrifugation was set at 4 °C. After centrifugation, the released oil was drained by inverting the tubes for 30–35 min. The comparison between the mass of the sample before and after a centrifugation cycle allowed the determination of the oil loss as a percentage of the initial oleogel mass [29].

#### 4.3.3. Oleogel Rheology

The method described by Wijarnprecha et al. [35], with slight modifications, and the Anton Paar MCR302 rheometer (Anton Paar, Graz, Austria) were used. Samples’ viscosity was measured at 20 s^−1^ using a parallel plate geometry (PP50) with a diameter of 50 mm. The baseplate was initially heated at 90 °C, and the samples were placed on it and then cooled to 1 °C/min from 90 to 4 °C (ramp linear). The distance between the baseplate and geometry was set at 0.5 mm and the normal force at 0 N. Frequency sweep tests were performed at 20 °C within the LVR at a strain of 0.01% using a frequency range of 0.01–100 Hz.

### 4.4. Applicability of Oleogels in Tender Dough Products

#### 4.4.1. Dough and Biscuits Preparation

A total of 4 prototype biscuits were made according to the manufacturing recipe for each experimental design: 100 g of wheat flour, 50 g of oleogel, 33.33 g of powdered sugar, and 17.66 g of egg. A control sample was performed where the oleogel was replaced with commercial baker’s margarine. In the initial phase, the raw materials were analyzed qualitatively, dosed, and homogenized manually for 10 min in order to obtain a homogeneous dough, with corresponding physicochemical properties, which was kept for 15 min at the refrigeration temperature (2–4 °C) in order to achieve rest. In the next phase, the dough for biscuits was rolled to a thickness of 4 mm and shaped into 3 cm discs and baked in a preheated electric oven at 175 °C for 15 min. After cooling to room temperature, the biscuits samples were packed in polypropylene bags and stored at room temperature (18–20 °C) for further analysis. The samples were coded as: dough/biscuit with oleogel with carnauba wax (D/B_CRW), dough/biscuit with oleogel with glycerol monostearate (D/B_GM), dough/biscuit with oleogel with mixture of *β*-sitosterol:beeswax (D/B_BS:BW), dough/biscuit with oleogel with mixture of *β*-sitosterol:lecithin (D/B_BS:LEC), and dough/biscuit with commercial margarine (D/B_MR).

#### 4.4.2. Dough Texture Analysis

The method consists in the deformation of the dough balls (10 mm height × 20 mm diameter) in two cycles, with the cylindrical compression probe TA25/1000 (Brookfield CT3 texture analyzer, Brookfield Engineering Labs, Middleboro, MA, USA) and the method described by Mert and Demirkesen [48], with slight modifications. The compression of the samples was performed at a deformation percentage of 75% from the sample surface, following the hardness, adhesiveness, resilience, cohesiveness, and springiness index. The dough was prepared before the analysis, stored at 4 °C for 15 min, and analyzed directly from this temperature.

#### 4.4.3. Dynamic Viscoelastic Measurement of Dough

The changes in the dynamic viscoelastic properties of dough samples by margarine replacement with oleogels were investigated using a MCR 302 rheometer (Anton Paar, Graz, Austria) and the method described by Jung et al. [38], with slight modifications using the parallel plate geometry (PP50). The dough was placed between the parallel plates and compressed to obtain a gap of 2 mm, and excess samples were trimmed. Samples were allowed to rest for 15 min at 4 °C to ensure the relaxation of stresses (from the homogenization of raw materials) and before performing the analysis for 5 min for the stress induced during loading. Frequency sweep tests with a constant shear strain of 0.01% were performed from 0.1 to 10 Hz at 25 °C. Storage (G′) and loss (G′′) moduli and the loss factor (tan δ) were recorded.

#### 4.4.4. Biscuits Texture Analysis

The texture analysis of the biscuits was performed according to the method used by Zhao et al. [15] with slight modifications, by compressing the samples (5 mm height × 30 mm diameter) with the acrylic blade TA7. The compression of the samples was performed in a single cycle, at a depth of 6 mm from the sample surface, with a speed of 1 mm/s. The Brookfield CT3 texture analyzer (Brookfield Engineering Labs, Middleboro, MA, USA) records the load (N) as a function of time (s). The parameters followed in the textural analysis of the tender dough biscuits were hardness and fracturability. After obtaining, the biscuits were stored in polypropylene bags, at 18–20 °C, being analyzed directly from this temperature.

### 4.5. Color Determination

The color measurement of oleogels, doughs, and biscuits was performed with the portable colorimeter NR200 (3NH, Shenzhen, China). The lightness L*, a* (−a greenness, +a redness), and b* (−b blueness, +b yellowness) color parameters were measured. The instrument performed an automatic calibration (L* = 0, a* = 0, and b* = 0). The values L*, a*, b* were provided by the instrument software. The color difference (ΔE) between margarine and oleogel samples was calculated as ΔE = √ (L*_R_ − L*_E_)^2^ +(a*_R_ − a*_E_)^2^ +(b*_R_ − b*_E_)^2^, where R refers to the margarine samples and E to the oleogel samples [37].

### 4.6. Statistical Analysis

The analyses were performed in duplicate, except for the color determination (four repetitions) and rheological analysis (one repetition). Differences were analyzed using Minitab software. One-way analysis of variance (ANOVA) and Tukey’s comparison test at a significance level of *p* < 0.05 were used. All results were presented as mean ± SD (standard deviation).

## Figures and Tables

**Table 2:** Textural properties and color parameters of dough samples.

Parameters	D_GM	D_CRW	D_BS:BW	D_BS:LEC	D_MR
**Texture profile analysis**					
Hardness (N)	41.84 ^B^ ± 2.41	92.49 ^A^ ± 10.40	29.27 ^BC^ ± 1.22	31.63 ^BC^ ± 2.12	21.80 ^C^ ± 1.41
Adhesiveness (mJ)	3.55 ^A^ ± 1.06	3.90 ^A^ ± 0.98	4.35 ^A^ ± 0.49	2.05 ^A^ ± 0.35	4.90 ^A^ ± 1.27
Resilience	0.01 ^A^ ± 0.00	0.02 ^A^ ± 0.01	0.01 ^A^ ± 0.00	0.01 ^A^ ± 0.00	0.01 ^A^ ± 0.00
Cohesiveness	0.08 ^B^ ± 0.01	0.07 ^B^ ± 0.02	0.10 ^AB^ ± 0.01	0.08 ^B^ ± 0.01	0.27 ^A^ ± 0.10
Springiness index	0.11 ^A^ ± 0.02	0.12 ^A^ ± 0.07	0.11 ^A^ ± 0.01	0.11 ^A^ ± 0.01	0.23 ^A^ ± 0.07
**Color**					
L*	66.04 ^A^ ± 1.39	58.03 ^B^ ± 3.15	65.43 ^A^ ± 3.31	70.28 ^A^ ± 0.91	68.01 ^A^ ± 2.56
a*	6.98 ^A^ ± 0.51	6.73 ^AB^ ± 0.70	6.12 ^AB^ ± 0.35	6.75 ^AB^ ± 0.58	5.70 ^B^ ± 0.19
b*	22.11 ^BC^ ± 0.50	20.89 ^C^ ± 0.72	20.89 ^C^ ± 0.57	22.87 ^AB^ ± 0.62	24.22 ^A^ ± 0.74
ΔE	3.16	10.57	4.23	2.84	-

Values are expressed as mean ± standard deviation. For each characteristic, identically superscript capital letters indicate no significant differences (*p* > 0.05) between samples. ΔE indicates color differences of the samples in comparison to D_MR.

**Table 3:** Textural properties and color parameters of biscuits.

Parameters	B_GM	B_CRW	B_BS:BW	B_BS:LEC	B_MR
**Texture profile analysis**					
Hardness (N)	20.97 ^B^ ± 0.39	13.20 ^CD^ ± 0.40	11.22 ^D^ ± 0.37	14.05 ^C^ ± 0.16	28.74 ^A^ ± 1.18
Fracturability (N)	20.97 ^B^ ± 0.39	3.87 ^E^ ± 0.88	11.22 ^D^ ± 0.37	14.05 ^C^ ± 0.16	28.74 ^A^ ± 1.18
**Color**					
L*	68.21 ^A^ ± 2.35	68.99 ^A^ ± 4.38	68.93 ^A^ ± 3.31	69.50 ^A^ ± 1.98	73.62 ^A^ ± 1.72
a*	13.40 ^BC^ ± 2.00	17.30 ^AB^ ± 3.41	18.18 ^A^ ± 1.94	16.12 ^ABC^ ± 1.42	12.50 ^C^ ± 1.10
b*	25.85 ^B^ ± 1.20	27.35 ^AB^ ± 2.33	29.53 ^A^ ± 0.62	29.08 ^A^ ± 0.61	28.77 ^A^ ± 0.85
ΔE	6.21	6.82	7.41	5.49	-

Values are expressed as mean ± standard deviation. For each characteristic, identically superscript capital letters indicate no significant differences (*p* > 0.05) between samples. ΔE indicates color differences in the samples in comparison to B_MR.
